# Advancements and Challenges in Neurosurgical Practice in India: Where Do We Stand?

**DOI:** 10.7759/cureus.37738

**Published:** 2023-04-17

**Authors:** Abhijit Ravindra Chandankhede, Snehal D Thombre

**Affiliations:** 1 Department of Neurosurgery, Shree Siddheshwar Multispeciality Hospital, Dhule, IND; 2 Department of Anaesthesiology, Institute of Medical Sciences and SUM Hospital, Bhubaneswar, IND

**Keywords:** rural neurosurgery, challenges in healthcare, advances in neurosurgery, global healthcare systems, neurosurgery

## Abstract

The challenges facing neurosurgical healthcare in India include accessibility, affordability, infrastructure, medical malpractice, and training and education. The lack of infrastructure and shortage of trained professionals are significant issues impacting the quality of care provided to patients. To address these challenges, there is a need for increased investment in facilities, expanding access to specialized equipment, increasing the number of trained staff, and improving the overall quality of healthcare facilities. Collaboration between government, private sector, and non-profit organizations is also necessary to ensure that patients receive comprehensive, high-quality care, regardless of their location or ability to pay. Additionally, addressing the shortage of trained neurosurgeons, neurologists, and neuroanesthesiologist in India is crucial to meet the growing demand for their services.

## Editorial

Neurosurgery is a specialized medical field that deals with the diagnosis, treatment, and rehabilitation of conditions affecting the nervous system, including the brain, spinal cord, and peripheral nerves. The field of neurosurgery has evolved significantly over the years, with new technologies and techniques enabling neurosurgeons to perform complex procedures with greater precision and improved outcomes.

In India, there has been a growing demand for neurosurgical care, with an increasing number of patients seeking treatment for conditions such as brain tumors, stroke, and traumatic brain injuries. However, there are still several challenges facing the neurosurgical community in India, including inadequate infrastructure, limited access to care, and a shortage of trained professionals.

Despite these challenges, the neurosurgical community in India is making significant strides in advancing the field and improving patient outcomes. New technologies and techniques are being developed and implemented, and there is a growing focus on improving access to care for patients in need.

While there have been significant improvements in the field of neurosurgical healthcare in India, there are still several areas where the country falls short. Some of the major challenges facing neurosurgical healthcare in India include:

1. Accessibility: Despite an increase in the number of healthcare providers, many people in India still lack access to quality neurosurgical care, especially in rural and remote areas. This is a significant barrier to receiving timely and appropriate treatment.

2. Affordability: The cost of neurosurgical care can be prohibitive for many Indians, and out-of-pocket expenses can be a significant burden for families who need medical treatment. This often results in patients delaying or foregoing necessary treatment.

3. Infrastructure: The infrastructure in many Indian hospitals and clinics is inadequate, with a shortage of trained staff, outdated equipment, and limited facilities. This can impact the quality of care provided to patients.

4. Medical malpractice: While there are many highly skilled neurosurgeons in India, there have been several cases of medical malpractice and negligence in recent years. This has eroded public trust in the healthcare system and highlights the need for better regulation and oversight.

5. Training and education: There is a shortage of trained neurosurgeons and healthcare professionals in India, which impacts the quality and availability of care. There is a need for more comprehensive training and education programs to meet the growing demand for neurosurgical healthcare services.

The current status of neurosurgical healthcare in India is a mixed bag. While there has been tremendous progress in the field, there are still many challenges that need to be addressed.

One of the biggest challenges facing neurosurgical healthcare in India is the lack of infrastructure. Many hospitals in India lack the necessary equipment and facilities to perform complex neurosurgical procedures. This has resulted in many patients having to travel to other countries for treatment. The inadequate facilities for neurosurgery in many Indian hospitals and clinics are a major concern for patients and healthcare professionals alike. The shortage of trained staff, outdated equipment, and limited operating rooms can have a significant impact on the quality of care provided to patients. In many hospitals and clinics, there is a shortage of trained neurosurgeons, anesthesiologists, and other healthcare professionals who specialize in neurosurgery. This shortage can result in longer wait times for patients, delayed or inadequate treatment, and worsened outcomes. Additionally, there is often a lack of support staff, such as nurses and technicians, who are trained to assist during neurosurgical procedures.

Outdated equipment is another issue that can impact the quality of care provided to patients. Many hospitals and clinics in India lack the necessary equipment for neurosurgery, such as advanced imaging techniques, monitoring equipment, and specialized operating rooms. The limited operating rooms in many hospitals and clinics can also pose a challenge for neurosurgery. Neurosurgical procedures often require specialized equipment and dedicated operating rooms to ensure the best possible outcomes. However, many hospitals and clinics in India do not have sufficient operating rooms, which can result in longer wait times for patients and delays in surgery.

To address these issues, there is a need for increased investment in facilities for neurosurgery in India. This includes expanding access to specialized equipment, increasing the number of trained staff, and improving the overall quality of healthcare facilities. Additionally, there is a need for greater collaboration between government, private sector, and non-profit organizations to ensure that patients receive comprehensive, high-quality care, regardless of their location or ability to pay.

Another challenge facing neurosurgical healthcare in India is the shortage of trained professionals. There are not enough neurosurgeons, neurologists, and neuroanesthesiologists in India to meet the growing demand for their services. This has resulted in long waiting lists for patients who require neurosurgical treatment. Neuroanesthesia is a specialized field of anesthesia that deals with patients undergoing neurosurgical procedures. While there have been significant improvements in neuroanesthesia practice in India in recent years, there are still several challenges that need to be addressed. Some of the major challenges facing neuroanesthesia practice in India include:

1. Shortage of trained professionals: There is a shortage of trained neuroanesthesiologists in India, which impacts the quality of care provided to patients. Many hospitals and clinics lack specialized staff with expertise in neuroanesthesia.

2. Lack of infrastructure: Many hospitals and clinics lack the necessary infrastructure to support neurosurgical procedures. This includes access to advanced imaging techniques, monitoring equipment, and specialized operating rooms.

3. Limited availability of medications: Many medications used in neuroanesthesia practice are not readily available in India, which can lead to delays or compromises in patient care.

4. Inadequate pain management: Pain management is an essential part of neurosurgical procedures, but there is a lack of specialized pain management services in many hospitals and clinics in India. This can result in patients experiencing unnecessary pain and discomfort.

5. Challenges in managing comorbidities: Many patients undergoing neurosurgical procedures have multiple comorbidities, such as hypertension, diabetes, or heart disease. Managing these conditions during surgery can be challenging, requiring specialized expertise and equipment.

To address these challenges, there is a need for increased investment in infrastructure and training for neuroanesthesiologists in India. This includes expanding access to specialized equipment, medications, and pain management services. Additionally, there is a need for increased collaboration between neuroanesthesiologists and other healthcare professionals involved in neurosurgical procedures to ensure that patients receive comprehensive, high-quality care.

Despite these challenges, there have been many positive developments in neurosurgical healthcare in India in recent years. One of the most significant developments has been the increased availability of minimally invasive procedures. These procedures involve smaller incisions and result in less pain, shorter hospital stays, and faster recovery times.

Another positive development has been the increasing use of advanced imaging technologies such as MRI and CT scans. These technologies have greatly improved the accuracy of diagnoses and have allowed for more precise surgical planning.

India has also made significant progress in the field of neurorehabilitation. There are now many specialized rehabilitation centers that offer comprehensive programs to help patients recover from neurological disorders. Neurorehabilitation is an essential part of the healthcare system in India, especially for individuals who have suffered from neurological disorders or injuries. The field of neurorehabilitation in India has made significant progress in recent years, with the establishment of specialized centers that offer comprehensive programs to help patients recover and rehabilitate.

These centers offer a range of services, including physical therapy, occupational therapy, speech and language therapy, and psychological counseling. They provide specialized care for individuals with conditions such as stroke, traumatic brain injury, spinal cord injury, and Parkinson's disease.

One of the most significant developments in the field of neurorehabilitation in India has been the increasing use of technology. Many rehabilitation centers now use virtual reality, robotics, and other technologies to help patients improve their motor and cognitive functions. These technologies have proven to be effective in helping patients recover and regain their independence. Another area of progress in neurorehabilitation in India is the increasing focus on community-based rehabilitation. Many rehabilitation centers now work closely with local communities to provide support and assistance to individuals with neurological conditions. This approach has been effective in improving the quality of life for patients and reducing the burden on their families. Despite these positive developments, there are still significant challenges facing the field of neurorehabilitation in India. One of the biggest challenges is the shortage of trained professionals. There is a need for more specialized therapists and rehabilitation professionals to meet the growing demand for their services [1].

Overall, neurorehabilitation in India has made significant progress in recent years, but there is still a long way to go. Continued investment in infrastructure, training, and technology will be essential to ensure that individuals with neurological conditions receive the care and support they need to recover and lead fulfilling lives.

In addition, India has also made significant strides in the development of telemedicine technologies. These technologies allow patients to receive remote consultations and follow-up care, which is especially important for patients who live in rural or remote areas [2].

Finally, there is a growing recognition of the need to improve access to neurosurgical care for patients across India. This includes increasing the number of trained professionals, expanding access to advanced technologies and equipment, and improving the quality of healthcare facilities in underserved areas. Through these efforts, more patients will be able to receive the care they need, regardless of their location or socioeconomic status.

Future of neurosurgery in India

The future of neurosurgery in India looks promising, with a growing emphasis on technology, innovation, and collaboration. Despite the challenges facing the field, there are several factors that point toward a brighter future for neurosurgical care in India.

One of the key drivers of change in the field of neurosurgery is technology. New advancements in surgical tools and techniques are allowing neurosurgeons to perform complex procedures with greater accuracy and precision. For example, the development of 3D printing technology is enabling neurosurgeons to create customized implants and surgical tools that are tailored to the unique needs of each patient. In addition, advances in robotics are enabling surgeons to perform procedures with greater precision and accuracy, reducing the risk of complications and improving patient outcomes.

The recent advances in the field of artificial intelligence have also paved a strong path toward the frequent use of artificial intelligence in managing clinical tasks, preoperative assessment, and diagnosis for neurosurgical patients. The patient-centric approach to the use of artificial intelligence for the automation of these tasks is proving helpful. However, the challenges include patient privacy, managing the data, and overreliance on artificial intelligence [3].

Another factor contributing to the future of neurosurgery in India is the growing emphasis on collaboration. As the field becomes more complex and specialized, there is a greater need for interdisciplinary collaboration between neurosurgeons, radiologists, anesthesiologists, and other healthcare professionals. This collaboration can lead to improved outcomes for patients, as well as greater efficiency and effectiveness in the delivery of care.

Conclusion

Even though there are still many challenges facing neurosurgical healthcare in India, there have also been many positive developments in recent years. With continued investment in infrastructure, training, and technology, India has the potential to become a world leader in the field of neurosurgery.
